# Behavioral performance and visual strategies during skill acquisition using a novel tool use motor learning task

**DOI:** 10.1038/s41598-018-32001-4

**Published:** 2018-09-13

**Authors:** T. J. Bosch, T. Hanna, K. A. Fercho, L. A. Baugh

**Affiliations:** 1 Basic Biomedical Sciences, University of South Dakota Sanford School of Medicine, 414 E Clark St, Vermillion, SD 57069 USA; 2 Center for Brain and Behavior Research, University of South Dakota, 414 E Clark St, Vermillion, SD 57069 USA

## Abstract

Habitual tool use is considered a hallmark of human evolution. One potential explanation for humanity’s advanced tool using abilities is that humans possess a unique neurobiological system that estimates efficient ways to manipulate objects with novel tools, resulting in rapid tool use motor learning. However, since humans regularly use a multitude of tools, examining these underlying processes is challenging. As such, we developed a tool use motor learning paradigm that utilizes a challenging tool (chopsticks) in order to accomplish a difficult behavioral task that requires extensive practice to continuously improve one’s proficiency. We then assessed the paradigm by recruiting participants with little experience using chopsticks and training them over the course of eight training sessions to grasp marbles with chopsticks and drop them in a cylindrical container. Throughout training, we examined behavioral performance and visual strategies to determine whether practicing the task resulted in outcomes congruent with traditional motor learning. Results show that performance increases in a logarithmic pattern and is accompanied by decreased confirmatory visual strategies. Since these findings are congruent with those seen in traditional motor learning, this paradigm is a novel method for use in future research examining the underlying mechanisms that mediate tool use motor learning.

## Introduction

Humanity’s advanced tool using capabilities are considered a hallmark of evolution and are thought to facilitate rapid advancements in technology^1^. However, studying the mechanisms that underlie tool use motor learning in humans is challenging due to the ease and regularity that humans use tools. In previous examinations of tool use motor learning, simple tools that require little practice to gain proficiency have been used to characterize the underlying mechanisms mediating the acquisition of skilled tool use^2,3^. As such, it is important to establish a tool use motor learning paradigm that utilizes a tool that is challenging to use and a behavioral task where one can continuously improve their performance through practice. In this study, we developed a behavioral task where individuals use chopsticks to grasp marbles and drop them in a cylindrical container. We then assessed behavioral performance and visual strategies in individuals who were naïve to the use of chopsticks while they performed the task over the course of eight behavioral training sessions.

Although multiple species possess the ability to use tools^4^, only humans possess an advanced technological culture which emphasizes their use and manufacture^1,5–7^. In addition to widespread use, humans are able to rapidly acquire the ability to skillfully use new tools^8^, whereas non-human primates undergo a significant trial and error period before acquiring the ability to use a new tool^9^. One potential explanation for humanity’s advanced tool using capabilities is that humans possess cognitive abilities that facilitate experience-independent tool action representation^5^. For example, a region of the left inferior parietal lobe (IPL) is consistently active in humans during tool use action observation^10,11^, whereas non-human primates do not exhibit activation in homologous regions of their brains during tool use action observation. These findings suggest that humans may possess a unique neurobiological system tuned to represent unfamiliar tools^12,13^. However, given how regularly and easily humans use tools, examining the tool use motor learning process is challenging. Previous research examining the neurobiological mechanisms underlying tool use motor learning have typically used simple tools to perform tasks that require little practice to gain proficiency^2,3^. As such, in order to gain a better understanding of these underlying mechanisms, it is important to establish a tool use motor learning paradigm that utilizes a tool that is challenging to use in order to accomplish a behavioral task that is also challenging and that one can continuously improve their performance through practice.

In traditional motor learning paradigms, skill acquisition while performing a novel motor action occurs over a logarithmic time scale^14,15^. In other words, when one begins performing a novel motor action, increases in performance occur rapidly. However, as one continues practicing the novel action, increased performance becomes progressively more difficult to achieve over time. Therefore, assessing whether performance during motor skill acquisition increases over a logarithmic time scale is an effective method for examining motor learning.

In addition to measuring overt performance during motor skill acquisition, another method for examining the motor learning process is by examining how visual strategies change as one learns a new motor skill^16,17^. Since the visual and motor systems are tightly linked^18–21^, increases in overt behavioral performance are often complemented by changes in covert visual strategies^17,22^. For example, as one learns to shoot a basketball for the first time, the initial stage of motor learning involves learning the new spatial relationship between one’s hand and the basketball. During this initial stage, the visual system is focused on exploring the novel spatial relationship, resulting in a high number of confirmatory visual strategies (i.e. fixations that are centered on the interaction between one’s body and the object or environment)^22,23^ and longer average fixation durations in order to extract as much visual information about the new relationship as possible^24^. In the case of shooting a basketball, this initial stage of motor learning would be primarily characterized by visual strategies that confirm effective interactions between the hand and the basketball. While these initial strategies do not manifest in high behavioral performance^25^, they are important for establishing new spatial relationships.

After this initial exploratory stage, individuals gradually shift their visual strategies from confirmatory to anticipatory (i.e. fixations that are centered on the desired goal of the task)^22^. These strategies are predictive in nature^19,24,26,27^ and are positively correlated with increases in behavioral performance^22,25,28,29^. To return to the basketball example, after one becomes familiar with the proper interaction between the hand and the ball, their visual strategies will shift to the basketball hoop and will lead the basketball to its desired location. In addition to increasing the percentage of anticipatory visual strategies, motor skill acquisition is also accompanied by shorter fixation durations^17^, indicating that less time is needed to extract the necessary visual information for performing the action. Finally, since visual information can be extracted from peripheral vision as well as foveal vision^27^, increases in anticipatory visual strategies may also be accompanied by increases in the distance that the anticipatory strategy leads the hand or object.

Therefore, in the current study, we sought to develop and test a challenging tool use motor learning paradigm that is characterized by continuous improvements in behavioral performance through practice and accompanied by observable shifts in visual strategies. To accomplish this goal, we recruited research subjects who were naïve to the use of chopsticks and trained them to use this tool to perform a challenging behavioral task: grasping a marble with the chopsticks, lifting the marble to the apex of a cylindrical container, dropping the marble into the container, and continuing this action as many times as possible over the course of multiple one-minute trials. Over the course of eight behavioral training sessions, we assessed their behavioral performance while they performed the task to determine whether they could continuously improve their skill. In addition, during the first and final training sessions, we obtained eye-tracking measurements during performance to determine whether changes in visual strategies that accompany traditional motor learning also occur during tool use motor learning.

We predicted that subjects would continuously improve their performance throughout training, as reflected by the number of successfully completed marble drops, indicating that the behavioral task was challenging enough that individuals could continue to refine and improve their skill through practice. Furthermore, we predicted that increases in performance may best be represented by a logarithmic function, suggesting that the tool use paradigm employed reflects traditional principles of motor learning. For ocular behavior, we predicted that confirmatory visual strategies would be most prominent during the first behavioral training session, indicating that individuals were initially exploring the new spatial relationship between their hand, the chopsticks, and the marble. In comparison, we hypothesized that during the final behavioral training session anticipatory visual strategies would be most prominent, indicating that individuals shifted their attention from exploring the new spatial relationship to leading the chopsticks to the final behavioral goal. In addition to the distribution of visual strategies, we also predicted that the vertical distance between the chopsticks and the final behavioral goal during anticipatory gaze strategies would increase after training. This finding would be indicative of individuals being able to lead the chopsticks to the final goal at a greater distance and relying less on visual information from their peripheral vision. We also predicted that the average duration of both anticipatory and confirmatory visual strategies would decrease after training, based on the assumption that individuals would need to extract less confirmatory visual information as they increased their ability to skillfully perform the task. Confirmation of these predictions would validate this paradigm as a method for examining the tool use motor learning process by extending the skill acquisition period and would support its future use in examining the underlying mechanisms that mediate rapid tool use motor learning in humans.

## Materials and Methods

### Participants

Twelve participants were recruited from the University of South Dakota (7 female, 5 male; mean age = 21.33 years; age range = 18–28 years). All participants were required to be right handed, as assessed using a modified Edinburgh handedness inventory^30^, with normal or corrected-to-normal vision. In addition, each participant’s prior experience using chopsticks was assessed using a subjective questionnaire requiring participants to rank their ability to skillfully use chopsticks (scale = 1–6; 6 indicating high proficiency with chopstick use; included if ranking ≤3/6). All participants provided written informed consent for their participation in accordance with the experimental procedures that were approved by the University of South Dakota Institutional Review Board. All experiments were performed in accordance with the relevant guidelines and regulations set forth by the Declaration of Helsinki.

### Experimental Procedure

Over the course of four consecutive weeks, participants performed eight behavioral training sessions (two sessions per week, at least one day between sessions) in which they were trained to perform a task that was aimed at improving their skill level using chopsticks. The task consisted of grasping a marble (diameter of marble = 16 mm) with a pair of bamboo chopsticks (length of chopsticks = 260 mm), lifting the marble to the apex of a cylindrical container (diameter of cylinder = 45 mm), and repeating the action as many times as possible over the course of ten trials (duration = 60 seconds per trial). Each participant completed the first five trials using training chopsticks with a loop for the index and middle fingers and a bridge connecting the two individual chopsticks. These training chopsticks ensured that each participant learned to perform the action in a similar manner but were not used to evaluate performance. Following the five trials using the training chopsticks, each participant performed the next five trials using traditional chopsticks. The number of successful marble drops during each one-minute trial was recorded manually by the experimenter and later confirmed through video recordings. During the first and final behavioral training session, eye tracking recordings were obtained to characterize each participant’s visual strategies at the beginning and end of behavioral training.

### Eye-Tracking Specifications

Eye tracking data was collected using a pair of SMI eye-tracking glasses and iView ETG 2.2 experimental software (SensoMotoric Instruments, Teltow, Germany) with binocular tracking (sampling rate = 30 Hz; gaze tracking range = 80° horizontal, 60° vertical; accuracy = 0.5°; scene camera resolution = 1280 × 960). Before beginning each recording, calibration was performed using points in the upper left, bottom left, and bottom right visual field. Following data collection, eye-tracking data was imported into BeGaze 3.4 eye-tracking analysis software (SensoMotoric Instruments, Teltow, Germany) and the time range for each of the five one-minute trials using the traditional chopsticks was manually recorded. All fixations within each trial were then identified using BeGaze’s default algorithm for detecting fixations and saccades^31^ (maximum dispersion = 100 pixels; minimum duration = 80 milliseconds) and exported.

### Visual Strategy Classification

**Figure 1:**
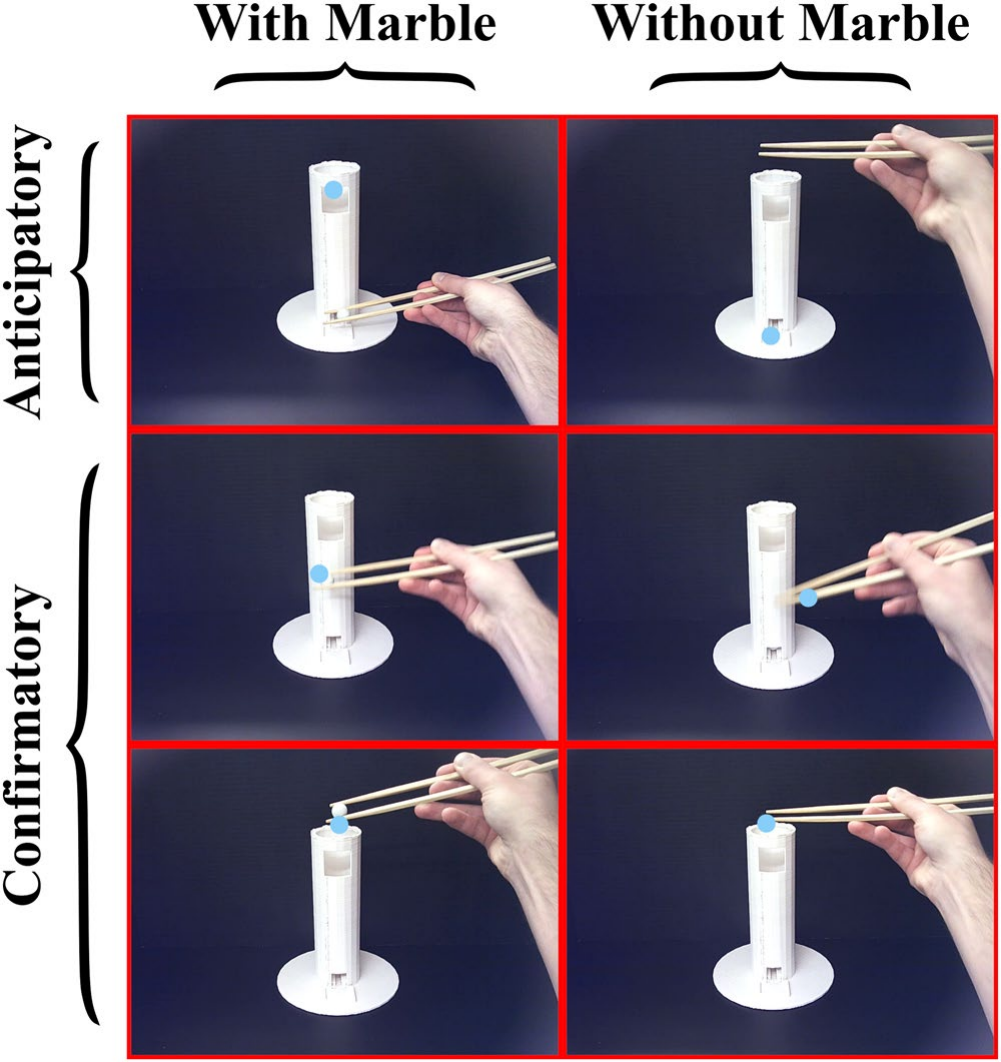
Examples of Visual Strategies – Anticipatory visual strategies were those where the vertical distance between the fixation point and the chopsticks tip was >36 mm. Confirmatory visual strategies were those where the vertical distance between the fixation point and the chopsticks tip was ≤36 mm. Visual strategies occurring while transporting the marble to the apex of the cylinder (with marble) were differentiated from those occurring after successfully dropping the marble into the cylinder and returning the chopsticks to the base of the cylinder (without marble). Blue dot = fixation point.

Following fixation export, each fixation was individually classified using custom LabVIEW software (National Instruments, Austin, TX) that identifies the corresponding video frame from the eye-tracker’s scene camera, and displays that frame to the experimenter for manual classification. Classification was initially based on whether the fixation occurred during transportation (i.e. transporting the marble with the chopsticks or transporting the chopsticks after successfully dropping the marble). Fixations that did not occur during transportation were removed from further analysis. In addition, fixations that were not experimentally-centered or goal-oriented (e.g. fixations outside the experimental setup) were also removed from further analysis. The length and slope of the cylinder was measured in each video frame in order to account for changes in each participant’s posture while performing the task. Next, the vertical distance between the chopsticks tip and fixation point was calculated relative to the length and slope of the cylinder. For example, if a participant sits close to the cylinder, the cylinder appears larger in the video frame compared to a participant sitting further away. Similarly, if a participant tilts their head during performance, the cylinder will appear rotated in the video frame compared to a participant who is not tilting their head. Therefore, calculating vertical distance between regions of interest (ROIs) at the center of the chopsticks tip and the center of the fixation point relative to the length and slope of the cylinder ensures that measurements are accurate regardless of the proximity or angle of each participant’s head relative to the cylinder. After calculating the vertical distance between ROIs, visual strategies were classified as anticipatory if the fixation ROI led the chopsticks ROI by >36 mm and confirmatory if the fixation ROI led the chopsticks ROI by ≤36 mm. The threshold of 36 mm was arbitrarily defined based on the diameter of one of the chopstick tips (4 mm) combined with the radius of the marble (8 mm) and multiplied by three to account for the dispersion algorithm utilized to define fixation points. After classifying visual strategies based on the distance between the fixation point and the chopsticks tip, anticipatory and confirmatory strategies were further classified based on whether they occurred while holding the marble with the chopsticks or while holding the chopsticks without the marble (See Fig. 1 for examples of each visual strategy).

### Data Analysis

#### Behavioral Data Analysis

Behavioral results were quantified as the number of successful marble drops per minute (MDPM) during the five trials using the traditional chopsticks. These metrics were then analyzed using a repeated measures ANOVA (critical α threshold = 0.05) across behavioral training sessions in SPSS (SPSS Statistics 20, IBM Corporation). To correct for multiple comparisons, subsequent pairwise comparisons between each training session were adjusted using the Holm-Bonferroni method. Effect sizes were calculated using Pearson’s *r* correlations.

In addition to MDPM, performance speed during separate phases of the task were determined to isolate tool learning (i.e. time to grasp, time to lift and drop) from phases of the task involving tool movement but no tool use (i.e. returning to the base of the cylinder after successfully dropping the marble). Two-tailed paired samples t-tests were used to determine changes between the first and final behavioral training session for each phase. Corrections for multiple comparisons and effect sizes were computed in an analogous manner to MDPM.

#### Eye Tracking Data Analysis

Eye tracking data was initially analyzed based on the percentage of anticipatory visual strategies compared to the percentage of confirmatory visual strategies between the first and final training sessions. These percentages were computed relative to the total number of visual strategies (anticipatory + confirmatory) and were used as input for a generalized estimating equations (GEE) analysis with visual strategy (anticipatory, confirmatory) and training session (first session, final session) as within-subjects factors. Since the percentages of anticipatory and confirmatory strategies for each session sum to 100%, only the main effect of visual strategy and the interaction between visual strategy and training session were examined.

While examining the percentages of anticipatory and confirmatory visual strategies reveals an indication of the overall distribution of each strategy compared to the total number of possible strategies, we were also interested in determining whether changes in this distribution were due to changes in the number of anticipatory strategies, changes in the number of confirmatory strategies, or a combination of both. To accomplish this, the total number of anticipatory and confirmatory visual strategies occurring in a single session was normalized based on the participant’s behavioral performance during that session (i.e. fixations per successful marble drop). These normalized values were then used as input for a two-way repeated measures ANOVA with visual strategy (anticipatory, confirmatory) and training session (first session, final session) as within-subjects factors.

Following these initial analyses, additional Spearman’s Rho correlations were performed comparing the percentage of anticipatory visual strategies and behavioral performance for both the first and final behavioral training session. Since any significant correlations between the percentage of anticipatory visual strategies and behavioral performance would be identical when comparing confirmatory visual strategies and behavioral performance – though reversed in directionality – only these initial correlations were performed.

In addition to determining how the distribution and number of anticipatory and confirmatory visual strategies change as a function of behavioral training, we were also interested in determining how these strategies change depending on the difficulty of the movement. For example, transporting the marble from the base of the cylinder to the apex of the cylinder requires one to consider both the relationship between the hand and the chopsticks as well as the relationship between the chopsticks and the marble. In contrast, transporting the chopsticks back to the base of the cylinder after successfully dropping the marble into the cylinder only requires one to consider the relationship between the hand and the chopsticks. With this in mind, we first determined the percentage of anticipatory and confirmatory strategies occurring while transporting the marble and those occurring after successfully dropping the marble relative to the total number of visual strategies in a particular session. These percentages were then used as input for a GEE analysis with visual strategy (anticipatory, confirmatory), difficulty (with marble, without marble), and training session (first session, final session) as within-subjects factors.

Next, we were interested in determining whether training and movement difficulty affected the average duration of each visual strategy. Therefore, the average duration of each fixation was used to perform a three-way repeated measures ANOVA with visual strategy (anticipatory, confirmatory), difficulty (with marble, without marble), and training session (first session, final session) as within-subject factors.

Finally, we examined whether training and movement difficulty affected the distance which an anticipatory visual strategy led the movement. To accomplish this, we used the average vertical distance between the chopsticks and the fixation point as input for a two-way repeated measures ANOVA with difficulty (with marble, without marble) and training session (first session, final session) as within-subjects factors.

For all eye tracking analyses, subsequent main effects and interactions between factors were followed up with Pearson’s *r* correlations to determine effect size.

## Results

### Behavioral Performance Results

**Figure 2:**
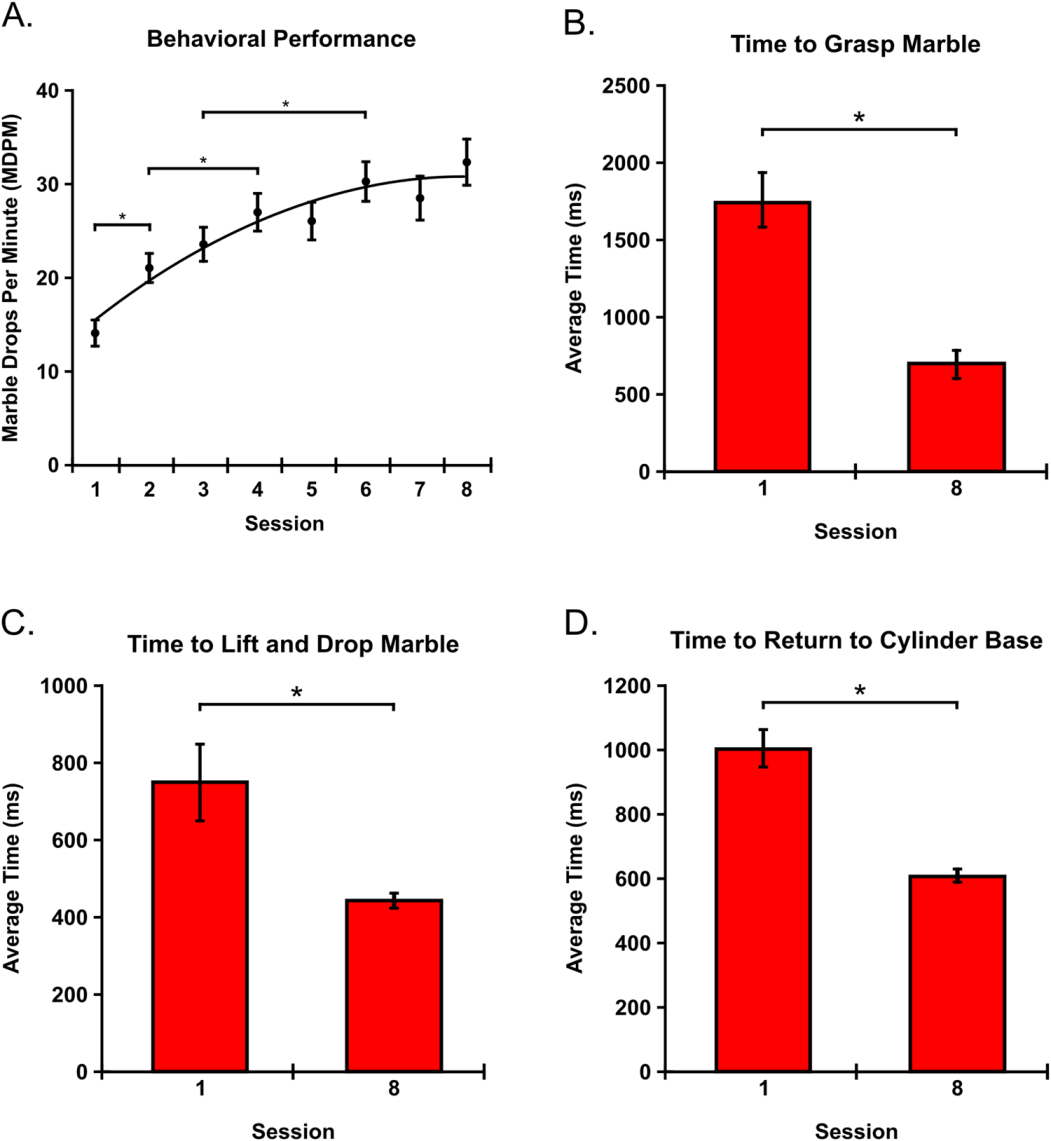
Behavioral Performance and Performance Speed. (**A**) Behavioral Performance. Participants continuously improved their performance throughout training. Significant increases were observed between the first and second, second and fourth, and third and sixth behavioral training sessions. (**B**–**D**) Performance Speed. Participants increased the speed with which they were able to grasp marbles from the base of the cylinder (**B**) lift marbles from the base to the apex of the cylinder (**C**) and return back to the cylinder base after successfully dropping the marble (**D**). *Denotes significance at the p < 0.05 level with Holm-Bonferroni corrections.

The mean familiarity for chopstick use reported was 1.83 on a six-point scale. For MDPM, we observed a main effect of training on behavioral performance [F (7, 77) = 23.404; p < 0.001] (Fig. 2A). Subsequent pairwise comparisons revealed significant increases in performance between session one [M = 14.217; SEM = 1.531] and session two [M = 21.117; SEM = 1.617] [p < 0.001; r = 0.75], session two and session four [M = 27.017; SEM = 2.058] [p = 0.002; r = 0.722], and between session three [M = 23.7; SEM = 1.895] and session six [M = 30.358; SEM = 2.229] [p = 0.001; r = 0.786].

Regarding performance speed, the average time to grasp the marble decreased between the first session [M = 1,750 ms; SEM = 175] and final session [M = 690 ms; SEM = 87] [p < 0.001; r = −0.031] (Fig. 2B). In addition, the average time to lift and drop the marble decreased between the first session [M = 748 ms; SEM = 99] and final session [M = 449 ms; SEM = 19] [p = 0.012; r = 0.063] (Fig. 2C). Finally, the average time to return to the base of the cylinder after successfully dropping the marble also decreased between the first session [M = 1,006 ms; SEM = 59] and final session [M = 606 ms; SEM = 22] [p < 0.001; r = −0.060] (Fig. 2D).

#### Eye-Tracking Results: Visual Strategy Distribution

**Figure 3:**
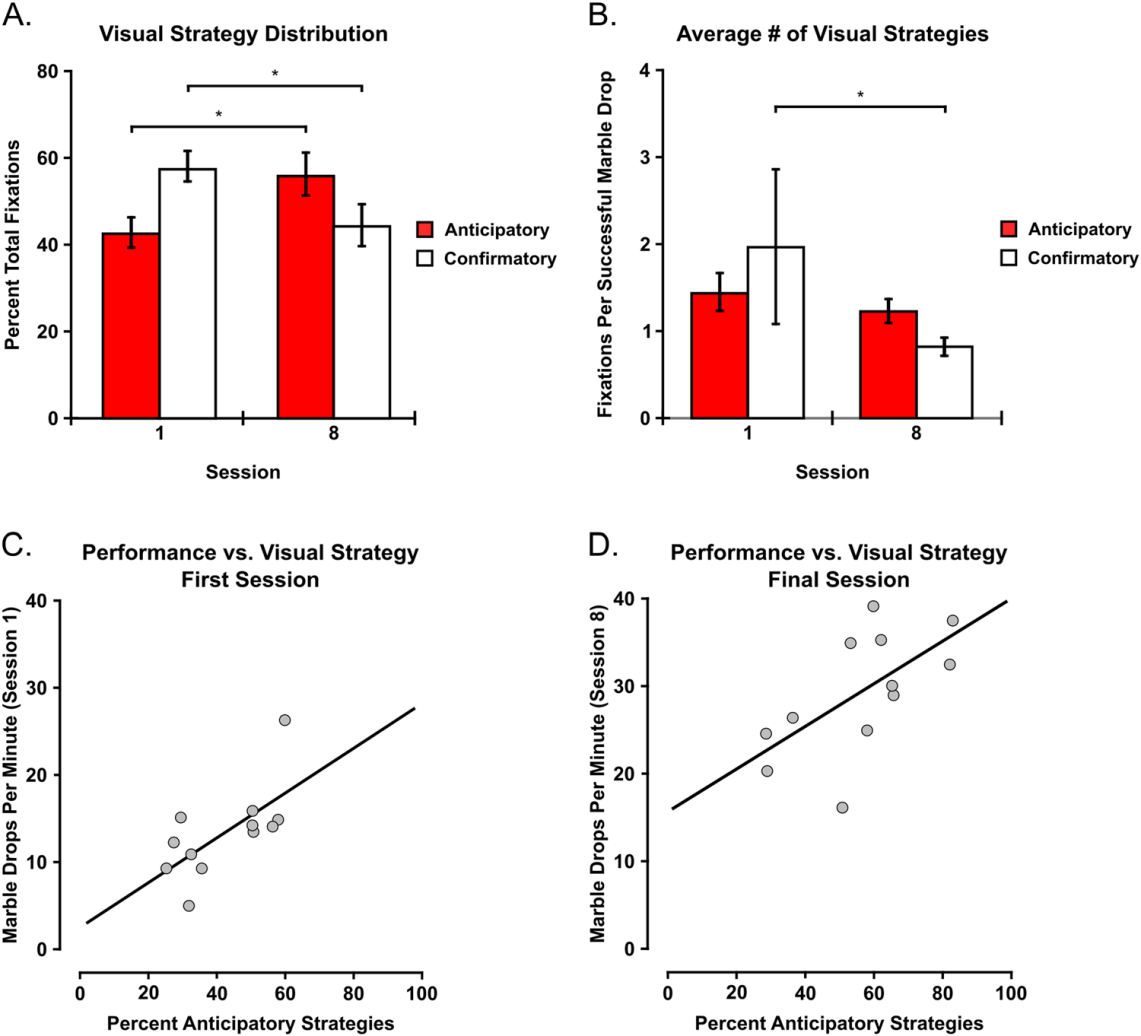
Visual Strategies and Behavioral Performance. (**A**) Visual Strategy Distribution. During the first behavioral training session, confirmatory visual strategies were more prominent than anticipatory strategies. This distribution was reversed during the final training session. (**B**) Average number of visual strategies per successful marble drop between the first and final training sessions. Significantly fewer confirmatory visual strategies per successful marble drop were observed after training. (**C**,**D**) Associations between anticipatory strategy distributions and behavioral performance for the first (**C**) and final (**D**) training sessions. *Denotes significance at the 0.05 level. Error bars indicate SEM. N = 12. Correlations computed using Spearman’s Rho.

The initial GEE analysis examining the distribution of visual strategies between the first and final training sessions revealed an interaction between visual strategy and training session [χ² = 5.015; p = 0.025] (Fig. 3A). Subsequent pairwise comparisons indicated that the percentage of anticipatory visual strategies for the first session [M = 42.367; SEM = 3.557] was lower compared to the final session [M = 55.761; SEM = 4.962] [p = 0.017; r = 0.466]. When comparing confirmatory strategies, the percentage of confirmatory visual strategies for the first session [M = 57.633; SEM = 3.557] was higher compared to the final session [M = 44.239; SEM = 4.962] [p = 0.017; r = 0.466]. No main effect of visual strategy [χ² = 0.065; p = 0.799] was observed.

Next, the two-way repeated measures ANOVA examining the number of fixations per successful marble drop revealed a main effect of training [F (1, 11) = 7.674; p = 0.018] with the first training session [M = 1.706; SEM = 0.257] displaying more fixations per marble drop than the final training session [M = 1.061; SEM = 0.060] [r = 0.512] (Fig. 3B). In addition, a significant interaction between visual strategy and training session was observed [F (1, 11) = 7.608; p = 0.019]. The subsequent pairwise comparison for anticipatory fixations per marble drop between the first [M = 1.446; SEM = 0.225] and final training session [M = 1.228; SEM = 0.141] revealed no significant differences [p = 0.204; r = 0.699]. However, confirmatory fixations per marble drop were greater for the first session [M = 1.966; SEM = 0.335] compared to the final session [M = 0.895; SEM = 0.104] [p = 0.013; r = 0.100]. No main effect of visual strategy was observed [F (1, 11) = 0.294; p = 0.598]. In addition, the Spearman’s correlations assessing the association between behavioral performance and the percentage of anticipatory visual strategies revealed significant associations between anticipatory gaze percentage and behavioral performance for both the first session [r_s_ = 0.586; p = 0.045] (Fig. 3C) and the final session [r_s_ = 0.664; p = 0.018] (Fig. 3D).

**Figure 4:**
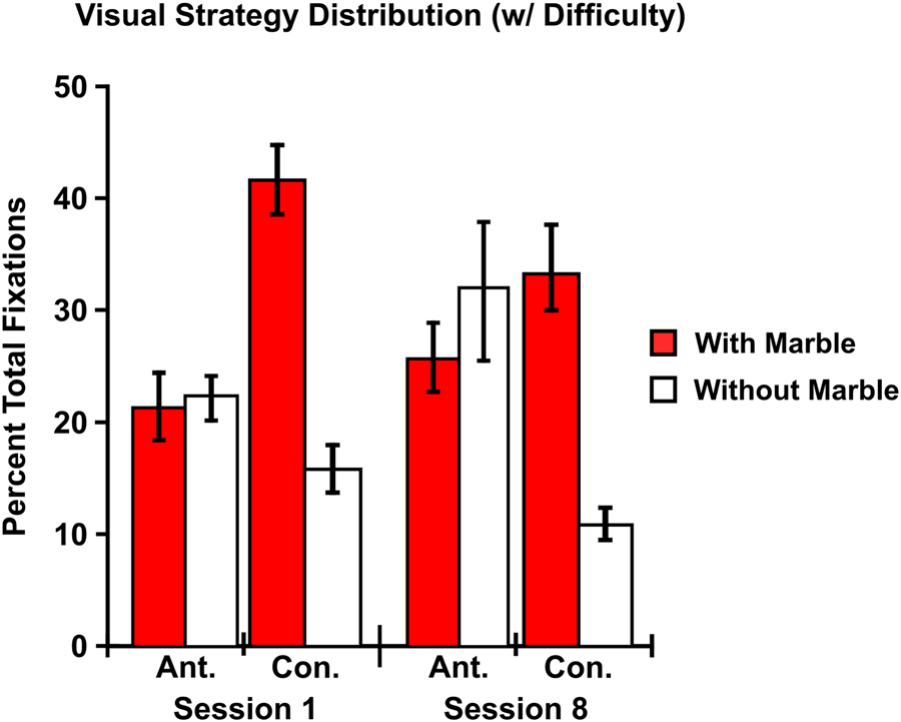
Visual Strategy Distribution (w/Difficulty) – Including difficulty in the GEE model resulted in a main effect of difficulty with strategies occurring while transporting the marble, particularly confirmatory strategies, being more prominent than those occurring without the marble. In addition, the reversal in distribution for anticipatory and confirmatory strategies after training (Fig. 3A) remains. Ant. = Anticipatory. Con. = Confirmatory. Error bars indicate SEM. N = 12.

After incorporating difficulty (with marble, without marble) into the model, the GEE analysis examining visual strategy distribution revealed a main effect of difficulty [χ² = 6.719; p = 0.010] with visual strategies occurring while transporting the marble [M = 30.008; SEM = 1.282] being more prominent than visual strategies occurring without the marble [M = 19.992; SEM = 1.282] (Fig. 4). In addition, an interaction between visual strategy and difficulty was observed [χ² = 8.013; p = 0.005]. No two-way interaction between training session and difficulty [χ² = 0.678; p = 0.410] or three-way interaction between visual strategy, training session, and difficulty was observed [χ² = 0.047; p = 0.829].

Follow-up pairwise comparisons for the two-way interaction between visual strategy and difficulty showed that confirmatory strategies occurring without the marble [M = 13.384; SEM = 1.534] were less prominent than confirmatory strategies occurring while transporting the marble [M = 37.552; SEM = 2.795] [p < 0.001; r = 0.381], anticipatory strategies occurring without the marble [M = 26.600; SEM = 3.510] [p = 0.023; r = −0.752], and anticipatory strategies occurring while transporting the marble [M = 22.464; SEM = 2.412] [p = 0.011; r = 0.018]. In addition, anticipatory strategies occurring while transporting the marble were less prominent than confirmatory strategies occurring while transporting the marble [p = 0.009; r = −0.524].

### Eye-Tracking Results: Visual Strategy Duration

**Figure 5:**
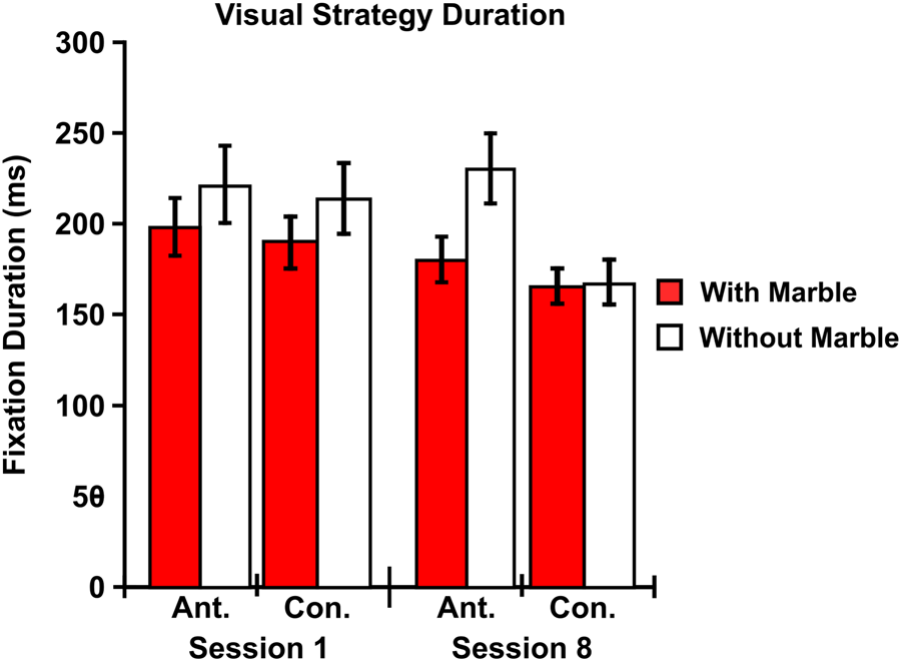
Visual Strategy Duration – No main effect of training on fixation duration was observed. However, the average duration of confirmatory fixations did decrease after behavioral training. In addition, fixations occurring without the marble, particularly anticipatory strategies, displayed a longer average duration. Ant. = Anticipatory. Con. = Confirmatory. Error bars indicate SEM. N = 12.

The three-way repeated measures ANOVA examining the average duration of visual strategies between the first and final training session revealed a main effect of difficulty [F (1, 11) = 5.719; p = 0.036] with strategies occurring while transporting the marble [M = 183.01; SEM = 10.910] displaying a shorter average duration compared to strategies occurring without the marble [M = 207.927; SEM = 13.954] [r = 0.704] (Fig. 5). Next, two-way interactions between visual strategy and difficulty [F (1, 11) = 5.064; p = 0.046] and between visual strategy and training session [F (1, 11) = 6.659; p = 0.026] were also observed. No main effects of visual strategy [F (1, 11) = 4.061; p = 0.069] or training session [F (1, 11) = 2.614; p = 0.134] were observed. In addition, no two-way interaction between difficulty and training session [F (1, 11) = 0.031; p = 0.864] or three-way interaction between visual strategy, difficulty, and training session [F (1, 11) = 4.109; p = 0.068] was observed.

Follow-up pairwise comparisons for the two-way interaction between visual strategy and difficulty showed that anticipatory strategies occurring without the marble [M = 224.741; SEM = 17.589] displayed a longer average duration than anticipatory strategies occurring while transporting the marble [M = 188.388; SEM = 13.010] [p = 0.004; r = 0.849], confirmatory strategies occurring without the marble [M = 191.114; SEM = 12.665] [p = 0.027; r = 0.694], and confirmatory strategies occurring while transporting the marble [M = 177.634; SEM = 11.103] [p = 0.022; r = 0.375]. In addition, follow-up pairwise comparisons for the two-way interaction between visual strategy and training showed that confirmatory strategies during the final session [M = 166.534; SEM = 7.812] displayed a shorter average duration than confirmatory strategies during the first session [M = 208.573; SEM = 17.954] [p = 0.029; r = 0.467], anticipatory strategies during the final session [M = 204.555; SEM = 13.817] [p = 0.007; r = 0.599], and anticipatory strategies during the first session [M = 208.574; SEM = 17.954] [p = 0.026; r = 0.499].

### Eye-Tracking Results: Anticipatory Strategy Distance

**Figure 6:**
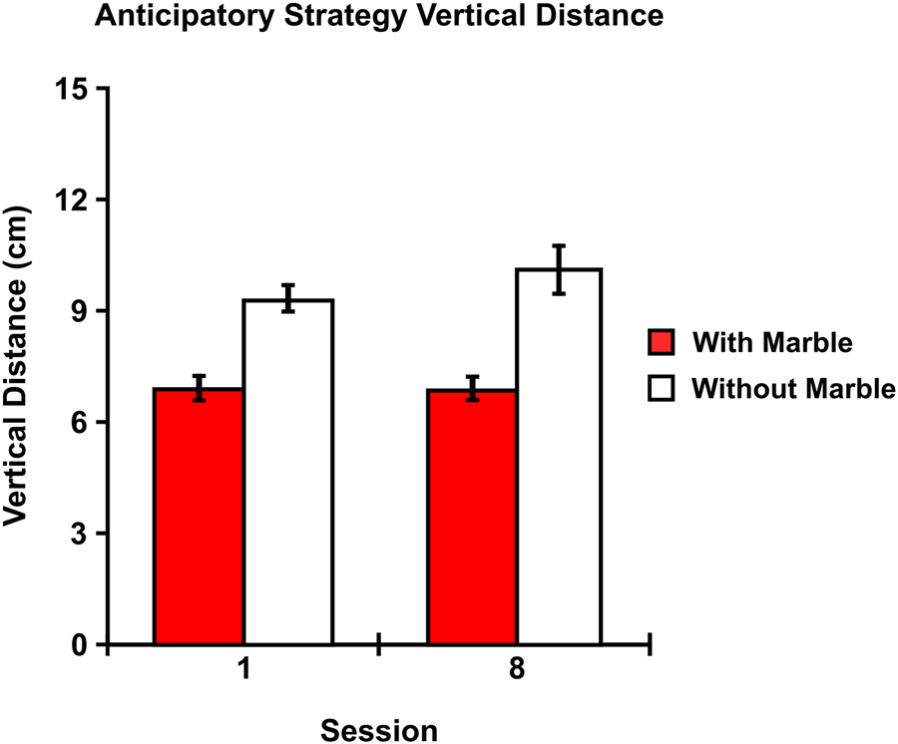
Anticipatory Strategy Vertical Distance – The average vertical distance for anticipatory strategies occurring without the marble was greater than anticipatory strategies occurring while transporting the marble. Error bars indicate SEM. N = 12.

Regarding distance, the two-way repeated measures ANOVA examining the average vertical distance between the chopsticks and the fixation point during anticipatory visual strategies revealed a main effect of difficulty [F (1, 11) = 22.183; p = 0.001] with strategies occurring without the marble [M = 9.654; SEM = 0.454] displaying a greater vertical distance between the tool and the fixation point compared to strategies occurring while transporting the marble [M = 6.857; SEM = 0.278] [r = 0.275] (Fig. 6). No main effect of training [F (1, 11) = 0.912; p = 0.360] or interaction between difficulty and training session [F (1, 11) = 0.952; p = 0.350] was observed.

## Discussion

The purpose of this study was to develop a novel tool use motor learning paradigm utilizing a challenging tool to perform a difficult behavioral task and examine whether individuals could continuously improve their performance on the task through practice. In addition, we were also interested in evaluating how changes in behavioral performance were associated with changes in visual strategies. We confirmed that individuals continuously improved their task performance through practice and that increased performance followed a logarithmic pattern, as evidenced by an overall effect of training on performance and significant increases over progressively greater periods of time. Concerning the visual behavior, we predicted that confirmatory visual strategies would be most prominent during the first training session, whereas anticipatory visual strategies would be most prominent during the final training session. This prediction was also confirmed, as we observed a reversal in the distribution of anticipatory and confirmatory visual strategies after behavioral training. Next, we predicted that the average vertical distance between the tool and the fixation during anticipatory visual strategies would increase after training. However, this prediction was not confirmed, as no significant differences in average vertical distance for anticipatory strategies between the first and final training sessions were observed. Finally, our prediction that the average duration of fixations for both anticipatory and confirmatory strategies would decrease after training was confirmed for confirmatory strategies. However, there was no difference in average duration of fixations for anticipatory strategies. These results are next discussed in the context of the changes in behavioral performance and visual strategies observed in the study.

### Behavioral Performance

In order to determine whether the tool use motor learning paradigm we developed could be used to examine the acquisition of skilled tool use, the behavioral task needed to be simple enough that participants could learn how to perform it but challenging enough that they did not maximize their performance after a single training session. While we did note a significant increase in performance after a single training session, performance continued to improve with practice. Specifically, performance increased between the second and fourth sessions as well as between the third and sixth sessions (Fig. 2A). Considering the fit of the logarithmic trend line to the data, additional practice would have likely revealed additional increases in performance, but these increases would become increasingly more difficult to achieve. Since the time scale for motor performance improvements observed in the present study is congruent with the well-established logarithmic time scale of performance improvements observed during motor learning^14,15^, we conclude that the paradigm developed in this study is sufficient for examining the tool use motor learning process in a longitudinal fashion.

### Visual Strategy Distribution

In addition to assessing whether behavioral performance changes in a manner reflective of traditional motor learning, we were also interested in examining whether visual strategies change throughout the learning process as well. Indeed, we observed a reversal in the distribution of anticipatory and confirmatory visual strategies as a function of training, with confirmatory strategies dominating the first training session and anticipatory strategies dominating the final training session (Fig. 3A). As stated in the introduction, when one first learns to perform a novel motor task, the initial visual strategy is to focus on learning and exploring the new spatial mapping between oneself and the novel object or environment^22^. In this study, that initial exploratory phase involved learning the sensorimotor constraints between the hand, the chopsticks, and the marble. Once individuals became more familiar with the behavioral task and improved their performance through practice, fewer cognitive resources were needed to explore the spatial relationship between the hand, the chopsticks, and the marble. As a result, predictive visual strategies intended to extract visual information about the tool’s final location became more prominent (Fig. 3A). This reversal in visual strategy distribution is congruent with previous research examining how visual strategies change as one becomes more familiar with the task^22^. Indeed, we also found that the percent of anticipatory strategies relative to the total number of fixations was positively associated with behavioral performance during both the first training session (Fig. 3C) as well as the final training session (Fig. 3D), a result consistent with previous findings regarding the positive association between anticipatory strategies and performance^25,28,29^.

Next, we determined whether the reversal in visual strategy distribution was driven by an increase in the number of anticipatory strategies, a decrease in the number of confirmatory strategies, or a combination of both. While there was a decrease in the average number of confirmatory visual strategies per marble drop after training, there was no difference in the average number of anticipatory visual strategies per marble drop (Fig. 3B). This finding isn’t necessarily surprising when one considers the nature of the task and the average number of anticipatory fixations per successful marble drop. That is, individuals had two movement goals during the task: transport the marble to the apex of the cylinder and return the chopsticks to the base of the cylinder afterwards. If individuals performed an anticipatory visual strategy to intake visual information about the final location of the tool, they should commit between one and two anticipatory fixations per successful marble drop. Regarding confirmatory visual strategies, individuals should commit fewer confirmatory fixations per successful marble drop as they become more familiar with the task and no longer need as much visual information about the interaction between the hand, the chopsticks, and the marble. While these findings are not necessarily unexpected, they do suggest that the lower percentage of confirmatory visual strategies was driving increased performance rather than the positive association with the percentage of anticipatory visual strategies. In other words, by committing fewer confirmatory fixations, individuals were able to enhance their speed and performance throughout practice.

Finally, after decomposing strategies into those that occurred while transporting the marble to the cylinder (with marble) and those that occurred while returning the chopsticks to the base of the cylinder (without marble), results showed that the distribution of visual strategies favored strategies occurring while transporting the marble more so than strategies occurring without the marble (Fig. 4). This finding is unsurprising, as the most challenging aspect of the task was most likely to be maintaining one’s grasp on the marble. As a result, individuals would need to alternate between anticipatory and confirmatory strategies so that they could maintain a proper grasp on the marble by directing a fixation from the goal of the task to the interaction during movement and obtaining more visual information.

### Visual Strategy Duration

Next, we also predicted that both anticipatory and confirmatory fixation durations would decrease after behavioral training, suggesting that less visual information was necessary in order to make an accurate movement. In contrast, we observed no main effect of training on fixation duration (Fig. 5). However, there was an overall decrease in the average duration of confirmatory fixations after behavioral training, suggesting that although similar amounts of visual information were necessary during anticipatory fixations, individuals no longer needed as much confirmatory information as they had previously needed when the task was still unfamiliar. In addition, fixations occurring without the marble, particularly anticipatory strategies without the marble, displayed a longer overall average duration compared to strategies occurring while transporting the marble. Similar to the effect of difficulty seen in visual strategy distribution, the need to alternate between anticipatory and confirmatory strategies to maintain a proper grasp on the marble likely resulted in each individual fixation being shorter in duration.

### Anticipatory Strategy Distance

In addition to assessing the general distribution and duration of anticipatory and confirmatory visual strategies, we also predicted that the average vertical distance between the tool and the fixation during anticipatory visual strategies would increase after training. This prediction was based on previous research indicating that visual information can be extracted from peripheral vision^27^. Specifically, we reasoned that if individuals were committing anticipatory visual strategies early in training, they might still be extracting visual information about the new spatial interaction between their hand, the chopsticks, and the marble. As a result, the average vertical distance between the fixation point and the chopsticks would be reduced compared to the final training session. However, we observed no difference in average vertical distance of anticipatory visual strategies between the first and final training sessions (Fig. 6). Instead, we found that the two movement goals displayed different average vertical distances. That is, anticipatory strategies that occurred as the chopsticks were returned to the base of the cylinder after a successful marble drop (without marble) displayed a greater average vertical distance between the tool and fixation point than anticipatory strategies that occurred while transporting the marble to the apex of the cylinder (with marble). Considering that differences in task demands and salience often result in different visual strategies^32^, the added difficulty and salience of holding the marble while moving the chopsticks likely contributed to this difference. A primary principal that guides interactions between the visual and motor system is that visual information is obtained in order to maximize motor accuracy and minimize motor error^33^. In this case, in order to minimize error due to the increased difficulty of grasping the marble, it is possible that the vertical distance of anticipatory strategies was reduced so that peripheral vision could intake more visual information about the status of the interaction between the tool and the marble.

## Conclusions

Overall, this study outlines a novel tool use motor learning paradigm involving a tool that is challenging to use (chopsticks) in order to perform a difficult task that one can continuously improve their performance through practice. We examined both behavioral performance and visual strategies in individuals who were naïve to the use of chopsticks over the course of eight behavioral training sessions where they learned how to use chopsticks to perform the task. Our results indicate that this tool use motor learning paradigm was not too challenging that individuals were unable to perform it but was difficult enough that extensive practice was required in order to dramatically improve one’s performance. In addition, our results demonstrate that the changes in visual strategies seen in traditional motor learning environments also occur when learning the present tool use task. Interestingly, we found that changes in confirmatory visual strategies were more prominent than changes in anticipatory visual strategies, suggesting that visual strategies become more efficient rather than more predictive throughout motor learning. While future research may seek to further validate this tool use motor learning paradigm by determining how the acquired tool manipulation skills translate to unpracticed contexts, at present it represents a novel method for examining the tool use motor learning process in a longitudinal fashion.

## Data Availability

The datasets generated during the current study are available from the corresponding author on reasonable request.
